# Poly[tetra-μ-aqua-diaqua­tetra­kis­[μ-(*E*)-2-nitro­cinnamato]tetra­rubidium]

**DOI:** 10.1107/S1600536811043406

**Published:** 2011-10-29

**Authors:** Graham Smith, Urs D. Wermuth

**Affiliations:** a Faculty of Science and Technology, Queensland University of Technology, GPO Box 2434, Brisbane, Queensland 4001, Australia

## Abstract

In the structure of the title compound, [Rb_4_(C_9_H_6_NO_4_)_4_(H_2_O)_6_]_*n*_, the asymmetric unit comprises four rubidium cations, two of which have an RbO_7_ coordination polyhedron with a monocapped distorted octa­hedral stereochemistry and two of which have a distorted RbO_6_ octa­hedral coordination. The bonding about both the seven-coordinate cations is similar, comprising one monodentate water mol­ecule together with three bridging water mol­ecules and three carboxyl­ate O-atom donors, two of which are bridging. The environments around the six-coordinate cations are also similar, comprising a monodentate nitro O-atom donor, a bridging water mol­ecule and four bridging carboxyl­ate O-atom donors [overall Rb—O range = 2.849 (2)–3.190 (2) Å]. The coordination leads to a two-dimensional polymeric structure extending parallel to (001), which is stabilized by inter­layer water O—H⋯O hydrogen-bonding associations to water, carboxyl and nitro O-atom acceptors, together with weak inter-ring π–π inter­actions [minimum ring centroid–centroid separation = 3.5319 (19) Å].

## Related literature

For the structures of some Rb complexes with aromatic carb­oxy­lic acids, see: Dinnebier *et al.* (2002 ▶); Wiesbrock & Schmidbaur (2003 ▶); Smith *et al.* (2007 ▶). For the structures of the two 2-nitro­cinnamic acid polymorphs, see: Schmidt (1964 ▶); Smith *et al.* (2006 ▶). For the structure of the Na salt of the acid, see: Smith & Wermuth (2009 ▶).
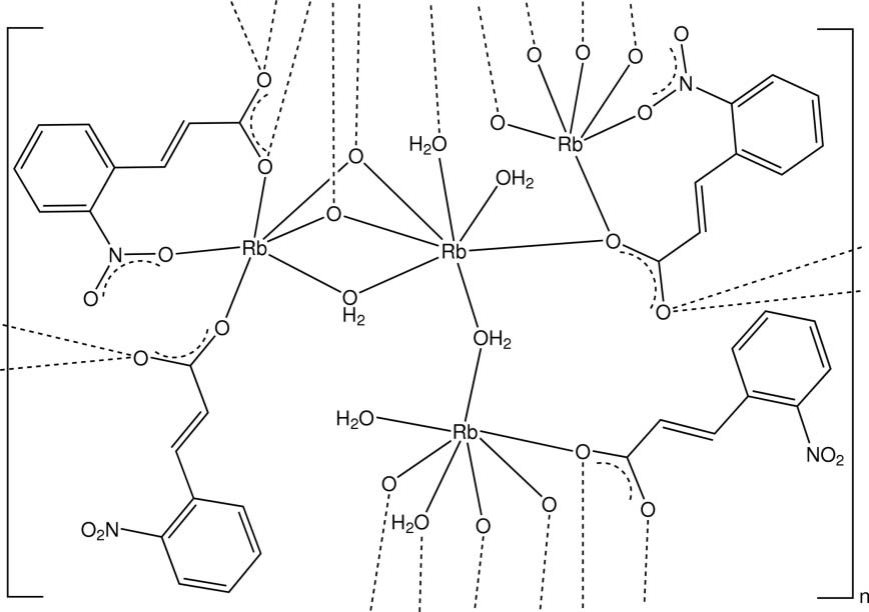


## Experimental

### 

#### Crystal data

[Rb_4_(C_9_H_6_NO_4_)_4_(H_2_O)_6_]
*M* *_r_* = 1218.57
Triclinic, 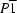

*a* = 7.02312 (14) Å
*b* = 7.77072 (15) Å
*c* = 41.1902 (8) Å
α = 89.5447 (15)°
β = 88.6733 (16)°
γ = 84.8679 (16)°
*V* = 2238.29 (8) Å^3^
*Z* = 2
Mo *K*α radiation
μ = 4.44 mm^−1^
*T* = 200 K
0.40 × 0.30 × 0.15 mm

#### Data collection

Oxford Diffraction Gemini-S CCD detector diffractometer
Absorption correction: multi-scan (*CrysAlis PRO*; Oxford Diffraction, 2010 ▶) *T*_min_ = 0.591, *T*_max_ = 0.980
26954 measured reflections
8812 independent reflections
6333 reflections with *I* > 2σ(*I*)
*R*_int_ = 0.036

#### Refinement

*R*[*F* ^2^ > 2σ(*F* ^2^)] = 0.039
*wR*(*F* ^2^) = 0.064
*S* = 1.05
8812 reflections
595 parameters
H-atom parameters constrained
Δρ_max_ = 0.50 e Å^−3^
Δρ_min_ = −0.53 e Å^−3^

### 

Data collection: *CrysAlis PRO* (Oxford Diffraction, 2010 ▶); cell refinement: *CrysAlis PRO*; data reduction: *CrysAlis PRO*; program(s) used to solve structure: *SHELXS97* (Sheldrick, 2008 ▶); program(s) used to refine structure: *SHELXL97* (Sheldrick, 2008 ▶) within *WinGX* (Farrugia, 1999 ▶); molecular graphics: *PLATON* (Spek, 2009 ▶); software used to prepare material for publication: *PLATON*.

## Supplementary Material

Crystal structure: contains datablock(s) global, I. DOI: 10.1107/S1600536811043406/wm2542sup1.cif

Structure factors: contains datablock(s) I. DOI: 10.1107/S1600536811043406/wm2542Isup2.hkl

Additional supplementary materials:  crystallographic information; 3D view; checkCIF report

## Figures and Tables

**Table 1:** Hydrogen-bond geometry (Å, °)

*D*—H⋯*A*	*D*—H	H⋯*A*	*D*⋯*A*	*D*—H⋯*A*
O1*W*—H11*W*⋯O13*B*^i^	0.87	1.94	2.795 (3)	167
O1*W*—H12*W*⋯O13*D*^i^	0.86	1.91	2.753 (3)	167
O2*W*—H21*W*⋯O4*W*^ii^	0.82	1.97	2.788 (3)	170
O2*W*—H22*W*⋯O14*C*^ii^	0.85	1.93	2.716 (3)	153
O3*W*—H31*W*⋯O14*D*^i^	0.91	1.80	2.695 (3)	169
O3*W*—H32*W*⋯O6*W*^iii^	0.88	1.86	2.728 (3)	170
O4*W*—H41*W*⋯O1*W*	0.84	2.02	2.852 (3)	178
O4*W*—H42*W*⋯O14*A*	0.84	1.91	2.758 (3)	180
O5*W*—H51*W*⋯O3*W*^iv^	0.94	1.82	2.734 (3)	163
O5*W*—H52*W*⋯O14*B*^v^	0.83	2.07	2.893 (3)	170
O6*W*—H61*W*⋯O13*C*^ii^	0.86	1.88	2.742 (3)	179
O6*W*—H62*W*⋯O13*A*	0.85	2.00	2.834 (3)	165

Symmetry codes: (i) 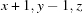; (ii) 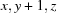; (iii) 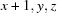; (iv) 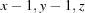; (v) 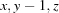.

**Fig. 1.**
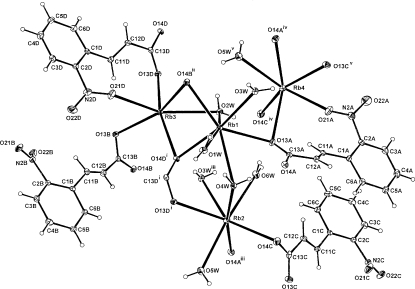
The molecular configuration and atom-numbering scheme for the coordination of the four rubidium cations in the title complex, with non-H atoms drawn as 40% probability ellipsoids. [Symmetry codes: (i) x, y, z -1; (ii) -x, -y, -z.]

**Fig. 2.**
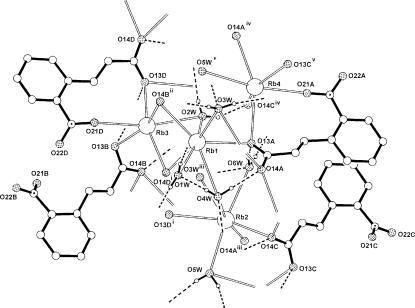
A view of a part of the structure showing structure extensions and intra-layer hydrogen bonds (as dashed lines). Non-associative H atoms were omitted. For symmetry codes, see Fig. 1 and Table 1.

**Fig. 3.**
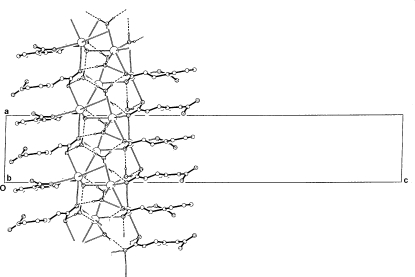
The two-dimensional polymeric structure viewed down the b axis of the unit cell, with hydrogen bonds shown as dashed lines.

## supplementary crystallographic information

### Comment

The structures of alkali metal complexes with aromatic carboxylic acids are of interest, particularly with the heavier metals Rb and Cs, because of their expanded and usually irregular coordination spheres, and their ability to form polymeric systems, commonly having carboxylate and water O bridges, *e.g.* rubidium salicylate with a [RbO_7_] coordination polyhedron (Dinnebier *et al.*, 2002), rubidium anthranilate monohydrate ([RbO_8_]) (Wiesbrock & Schmidbaur, 2003), or rubidium 5-sulfosalicylate 1.33 hydrate ([RbO_7_]) (Smith *et al.*, 2007).

We obtained crystals of the title compound [Rb_4_(C_9_H_6_NO_4_)_4_(H_2_O)_6_]_n_ from the reaction of *trans*-4-nitrocinnamic acid with rubidium hydroxide and the structure is reported here. There is only one example of a structure of an alkali metal complex with this ligand, sodium *trans*-2-nitrocinnamate dihydrate, a one-dimensional coordination polymer (Smith & Wermuth, 2009). In the structure of the title compound, the asymmetric unit comprises four rubidium cations, two of which are associated with [RbO_7_] cordination polyhedra [Rb1—O, 2.849 (2)–3.106 (2) Å; Rb2—O, 2.908 (2)–3.132 (2) Å] and two [RbO_6_] coordination polyhedra [Rb3—O, 2.901 (2)–2.975 (2) Å; Rb4—O, 2.883 (2)–3.190 (2) Å] (Fig. 1). The stereochemistry about both 7-coordinate cations is monocapped distorted octahedral while it is distorted octahedral for the 6-coordinate cations. The coordination spheres of both Rb1 and Rb2 comprise one monodentate water (O1*W* and O6*W*, respectively) and three bridging water molecules together with three carboxyl *O*-donors, two of which are bridging. The coordination spheres about both Rb3 and Rb4 are also similar in having the same distribution of donor types: a monodentate nitro O atom [O21*D* (Rb3) and O21*A* (Rb4)], one water and four carboxylate donors, all bridging. The overall complex has apparent pseudo-twofold rotational symmetry but no reasonable higher crystallographic symmetry could be invoked for the structure.

The two-dimensional polymeric structure (Figs. 2, 3) is expanding parallel to (001) and is stabilized by intra-layer and intermolecular water O—H···O hydrogen-bonding interactions to water, carboxyl and nitro *O*-acceptors (Table 1). Present also in the structure are inter-ring π—π interactions [minimum ring centroid–centroid separation, 3.5319 (19) Å]. The four 2-nitrocinnamate anions have minor conformational variations: the comparative side chain torsion angles (C2–C1–C11–C12), -146.6 (4), -157.3 (3), 147.8 (4) and -150.3 (4)° for *A*–*D* and the nitro group torsion angles (C1–C2–N2–O22), 161.9 (4), -146.7 (3), 150.4 (3) and -146.3 (3)° for *A*–*D*. This stereochemistry is similar to that found in both polymorphs of the parent acid (Schmidt, 1964; Smith *et al.*, 2006).

### Experimental

The title compound was synthesized by heating together under reflux for 15 minutes, 2 mmol of *trans*-cinnamic acid with 1 mmol of rubidium hydroxide in 50 ml of a 1:9 ethanol–water mixture. After concentration to *ca* 30 ml, partial room temperature evaporation of the hot-filtered solution gave colourless flat prisms of the title compound, from which a suitable specimen was cleaved for the X-ray analysis. The crystals were found to deteriorate when exposed to air.

### Refinement

The water H atoms were located in difference-Fourier syntheses but in the final cycles of refinement their positional parameters were constrained with their isotropic displacement parameters allowed to ride on the parent O atom, with *U*_iso_(H) = 1.2*U*_eq_(O). Other hydrogen atoms were included in calculated positions with C—H = 0.95 Å, also with *U*_iso_(H) = 1.2*U*_eq_(C).

### Crystal data

**Table:**

[Rb_4_(C_9_H_6_NO_4_)_4_(H_2_O)_6_]	*Z* = 2
*M**_r_* = 1218.57	*F*(000) = 1208
Triclinic, *P*1	*D*_x_ = 1.808 Mg m^−^^3^
Hall symbol: -P 1	Mo *K*α radiation, λ = 0.71073 Å
*a* = 7.02312 (14) Å	Cell parameters from 10977 reflections
*b* = 7.77072 (15) Å	θ = 3.2–28.7°
*c* = 41.1902 (8) Å	µ = 4.44 mm^−^^1^
α = 89.5447 (15)°	*T* = 200 K
β = 88.6733 (16)°	Plate, colourless
γ = 84.8679 (16)°	0.40 × 0.30 × 0.15 mm
*V* = 2238.29 (8) Å^3^	

### Data collection

**Table:**

Oxford Diffraction Gemini-S CCD detector diffractometer	8812 independent reflections
Radiation source: Enhance (Mo) X-ray source	6333 reflections with *I* > 2σ(*I*)
graphite	*R*_int_ = 0.036
Detector resolution: 16.077 pixels mm^-1^	θ_max_ = 26.0°, θ_min_ = 3.2°
ω scans	*h* = −8→8
Absorption correction: multi-scan (*CrysAlis PRO*; Oxford Diffraction, 2010)	*k* = −9→9
*T*_min_ = 0.591, *T*_max_ = 0.980	*l* = −50→50
26954 measured reflections	

### Refinement

**Table:**

Refinement on *F*^2^	Primary atom site location: structure-invariant direct methods
Least-squares matrix: full	Secondary atom site location: difference Fourier map
*R*[*F*^2^ > 2σ(*F*^2^)] = 0.039	Hydrogen site location: inferred from neighbouring sites
*wR*(*F*^2^) = 0.064	H-atom parameters constrained
*S* = 1.05	*w* = 1/[σ^2^(*F*_o_^2^) + (0.0237*P*)^2^where *P* = (*F*_o_^2^ + 2*F*_c_^2^)/3
8812 reflections	(Δ/σ)_max_ = 0.001
595 parameters	Δρ_max_ = 0.50 e Å^−^^3^
0 restraints	Δρ_min_ = −0.53 e Å^−^^3^

### Special details

**Table:**

Geometry. Bond distances, angles *etc*. have been calculated using the rounded fractional coordinates. All su's are estimated from the variances of the (full) variance-covariance matrix. The cell e.s.d.'s are taken into account in the estimation of distances, angles and torsion angles
Refinement. Refinement of *F*^2^ against ALL reflections. The weighted *R*-factor *wR* and goodness of fit *S* are based on *F*^2^, conventional *R*-factors *R* are based on *F*, with *F* set to zero for negative *F*^2^. The threshold expression of *F*^2^ > σ(*F*^2^) is used only for calculating *R*-factors(gt) *etc*. and is not relevant to the choice of reflections for refinement. *R*-factors based on *F*^2^ are statistically about twice as large as those based on *F*, and *R*- factors based on ALL data will be even larger.

### Fractional atomic coordinates and isotropic or equivalent isotropic displacement parameters (Å^2^)

**Table:**

	*x*	*y*	*z*	*U*_iso_*/*U*_eq_	
Rb1	0.94219 (5)	0.91218 (4)	0.26938 (1)	0.0212 (1)	
Rb2	0.44642 (5)	0.56962 (4)	0.23154 (1)	0.0234 (1)	
Rb3	0.55354 (5)	1.25576 (4)	0.31407 (1)	0.0245 (1)	
Rb4	1.05994 (5)	1.23024 (4)	0.18677 (1)	0.0283 (1)	
O1W	1.0765 (3)	0.6063 (3)	0.30690 (6)	0.0309 (9)	
O2W	0.7203 (3)	1.2134 (3)	0.24727 (6)	0.0257 (8)	
O3W	1.3448 (3)	0.9459 (3)	0.25016 (6)	0.0279 (8)	
O4W	0.8527 (3)	0.5397 (3)	0.25226 (6)	0.0253 (8)	
O5W	0.2304 (3)	0.2898 (3)	0.25649 (6)	0.0357 (9)	
O6W	0.5407 (3)	0.9000 (3)	0.19248 (6)	0.0327 (9)	
O13A	0.9422 (3)	0.8720 (3)	0.20024 (6)	0.0278 (8)	
O13B	0.1694 (3)	1.3122 (3)	0.34482 (6)	0.0269 (9)	
O13C	0.4344 (3)	0.1885 (3)	0.15619 (6)	0.0310 (9)	
O13D	0.4657 (3)	1.6262 (3)	0.30273 (6)	0.0276 (8)	
O14A	1.0819 (3)	0.6021 (3)	0.19896 (6)	0.0240 (8)	
O14B	−0.0257 (3)	1.2087 (3)	0.30933 (6)	0.0263 (8)	
O14C	0.6508 (3)	0.3115 (3)	0.18492 (6)	0.0328 (9)	
O14D	0.5749 (3)	1.8879 (3)	0.30129 (6)	0.0262 (8)	
O21A	0.9548 (5)	1.2102 (3)	0.11758 (8)	0.0596 (13)	
O21B	0.0040 (4)	1.1619 (3)	0.44788 (7)	0.0485 (10)	
O21C	0.4963 (5)	0.3392 (3)	0.05074 (7)	0.0547 (13)	
O21D	0.6288 (4)	1.2701 (3)	0.38398 (7)	0.0417 (10)	
O22A	0.9983 (6)	1.2938 (4)	0.06947 (8)	0.0849 (16)	
O22B	0.1989 (4)	1.0405 (3)	0.48277 (7)	0.0500 (11)	
O22C	0.2977 (4)	0.4607 (4)	0.01645 (7)	0.0518 (11)	
O22D	0.4952 (4)	1.1955 (3)	0.42886 (7)	0.0549 (11)	
N2A	0.9558 (4)	1.1848 (4)	0.08879 (9)	0.0326 (11)	
N2B	0.1058 (5)	1.0377 (4)	0.45818 (8)	0.0307 (11)	
N2C	0.3938 (5)	0.4642 (4)	0.04070 (8)	0.0330 (11)	
N2D	0.5696 (5)	1.3004 (4)	0.41170 (9)	0.0325 (11)	
C1A	0.9166 (4)	0.8696 (4)	0.09601 (8)	0.0186 (11)	
C1B	0.0955 (4)	0.8707 (4)	0.40683 (8)	0.0181 (11)	
C1C	0.4229 (4)	0.6317 (4)	0.09139 (8)	0.0173 (11)	
C1D	0.5767 (4)	1.6189 (4)	0.40592 (8)	0.0197 (11)	
C2A	0.9142 (5)	1.0156 (4)	0.07599 (9)	0.0223 (11)	
C2B	0.1075 (5)	0.8722 (4)	0.44044 (8)	0.0189 (11)	
C2C	0.3957 (5)	0.6294 (4)	0.05795 (8)	0.0200 (11)	
C2D	0.5912 (5)	1.4718 (4)	0.42573 (9)	0.0215 (11)	
C3A	0.8869 (5)	1.0081 (5)	0.04283 (9)	0.0309 (12)	
C3B	0.1138 (5)	0.7251 (4)	0.45964 (9)	0.0262 (12)	
C3C	0.3784 (5)	0.7768 (4)	0.03888 (9)	0.0285 (12)	
C3D	0.6245 (5)	1.4756 (5)	0.45856 (9)	0.0289 (12)	
C4A	0.8646 (5)	0.8536 (5)	0.02851 (9)	0.0351 (14)	
C4B	0.1017 (5)	0.5681 (4)	0.44488 (9)	0.0291 (14)	
C4C	0.3917 (5)	0.9349 (4)	0.05312 (10)	0.0297 (14)	
C4D	0.6441 (5)	1.6309 (5)	0.47308 (9)	0.0342 (14)	
C5A	0.8730 (5)	0.7050 (5)	0.04737 (9)	0.0311 (12)	
C5B	0.0846 (5)	0.5598 (4)	0.41148 (9)	0.0250 (11)	
C5C	0.4179 (5)	0.9423 (4)	0.08630 (10)	0.0285 (14)	
C5D	0.6280 (5)	1.7808 (5)	0.45456 (9)	0.0314 (14)	
C6A	0.8985 (5)	0.7140 (4)	0.08026 (9)	0.0241 (12)	
C6B	0.0848 (4)	0.7098 (4)	0.39309 (9)	0.0222 (12)	
C6C	0.4306 (4)	0.7938 (4)	0.10475 (9)	0.0231 (11)	
C6D	0.5926 (5)	1.7743 (4)	0.42188 (9)	0.0257 (12)	
C11A	0.9285 (5)	0.8678 (4)	0.13174 (8)	0.0197 (11)	
C11B	0.1094 (5)	1.0249 (4)	0.38621 (8)	0.0208 (11)	
C11C	0.4337 (5)	0.4762 (4)	0.11246 (8)	0.0202 (11)	
C11D	0.5346 (5)	1.6215 (4)	0.37112 (9)	0.0219 (11)	
C12A	1.0164 (5)	0.7420 (4)	0.14909 (9)	0.0229 (11)	
C12B	0.0410 (5)	1.0461 (4)	0.35657 (8)	0.0244 (11)	
C12C	0.5385 (5)	0.4579 (4)	0.13842 (9)	0.0250 (12)	
C12D	0.5965 (5)	1.7343 (4)	0.35014 (9)	0.0257 (12)	
C13A	1.0123 (5)	0.7401 (4)	0.18519 (9)	0.0200 (11)	
C13B	0.0642 (5)	1.2009 (4)	0.33608 (8)	0.0192 (11)	
C13C	0.5404 (5)	0.3069 (4)	0.16126 (9)	0.0248 (12)	
C13D	0.5417 (5)	1.7488 (4)	0.31544 (8)	0.0205 (11)	
H3A	0.88360	1.11070	0.03010	0.0370*	
H3B	0.12630	0.73220	0.48250	0.0310*	
H3C	0.35750	0.76910	0.01630	0.0340*	
H3D	0.63370	1.37180	0.47100	0.0350*	
H4A	0.84360	0.84810	0.00590	0.0420*	
H4B	0.10510	0.46540	0.45760	0.0340*	
H4C	0.38310	1.03750	0.04040	0.0360*	
H4D	0.66860	1.63560	0.49560	0.0410*	
H5A	0.86110	0.59640	0.03750	0.0370*	
H5B	0.07280	0.45220	0.40130	0.0300*	
H5C	0.42710	1.05080	0.09640	0.0340*	
H5D	0.64150	1.88890	0.46450	0.0380*	
H6A	0.90410	0.61040	0.09270	0.0290*	
H6B	0.07730	0.70200	0.37020	0.0270*	
H6C	0.44530	0.80260	0.12750	0.0280*	
H6D	0.57850	1.87920	0.40980	0.0310*	
H11A	0.86840	0.96450	0.14310	0.0240*	
H11B	0.17260	1.11690	0.39490	0.0250*	
H11C	0.36050	0.38420	0.10690	0.0240*	
H11D	0.45810	1.53680	0.36320	0.0260*	
H12A	1.08530	0.64860	0.13800	0.0270*	
H12B	−0.02690	0.95700	0.34800	0.0290*	
H12C	0.61860	0.54640	0.14300	0.0300*	
H12D	0.68230	1.81190	0.35770	0.0310*	
H11W	1.0870	0.5170	0.3200	0.0370*	
H12W	1.1940	0.6300	0.3060	0.0370*	
H21W	0.7626	1.3064	0.2511	0.0310*	
H22W	0.6990	1.2086	0.2270	0.0310*	
H31W	1.4325	0.9193	0.2656	0.0340*	
H32W	1.4051	0.9443	0.2312	0.0340*	
H41W	0.9200	0.5600	0.2680	0.0300*	
H42W	0.9230	0.5590	0.2360	0.0300*	
H51W	0.2630	0.1750	0.2500	0.0430*	
H52W	0.1550	0.2800	0.2720	0.0430*	
H61W	0.5080	0.9900	0.1810	0.0390*	
H62W	0.6580	0.9100	0.1960	0.0390*	

### Atomic displacement parameters (Å^2^)

**Table:**

	*U*^11^	*U*^22^	*U*^33^	*U*^12^	*U*^13^	*U*^23^
Rb1	0.0262 (2)	0.0171 (2)	0.0204 (2)	−0.0019 (1)	0.0002 (2)	−0.0002 (2)
Rb2	0.0249 (2)	0.0198 (2)	0.0253 (2)	0.0007 (2)	−0.0048 (2)	−0.0018 (2)
Rb3	0.0277 (2)	0.0211 (2)	0.0246 (2)	−0.0010 (2)	0.0015 (2)	−0.0025 (2)
Rb4	0.0295 (2)	0.0267 (2)	0.0293 (2)	−0.0062 (2)	−0.0006 (2)	−0.0025 (2)
O1W	0.0374 (16)	0.0230 (13)	0.0327 (16)	−0.0024 (11)	−0.0112 (13)	0.0077 (12)
O2W	0.0365 (15)	0.0207 (13)	0.0206 (14)	−0.0041 (11)	−0.0079 (11)	0.0017 (11)
O3W	0.0296 (15)	0.0294 (14)	0.0244 (15)	−0.0006 (11)	0.0013 (12)	−0.0019 (11)
O4W	0.0239 (14)	0.0321 (14)	0.0205 (15)	−0.0067 (11)	0.0000 (11)	−0.0007 (11)
O5W	0.0396 (16)	0.0243 (14)	0.0410 (18)	0.0054 (11)	0.0109 (13)	0.0038 (13)
O6W	0.0340 (16)	0.0355 (15)	0.0281 (16)	−0.0022 (12)	0.0020 (12)	0.0073 (12)
O13A	0.0419 (16)	0.0232 (13)	0.0177 (15)	0.0012 (11)	−0.0007 (12)	−0.0018 (11)
O13B	0.0371 (16)	0.0263 (14)	0.0193 (15)	−0.0130 (12)	−0.0037 (12)	−0.0003 (11)
O13C	0.0326 (16)	0.0296 (14)	0.0321 (17)	−0.0088 (12)	−0.0080 (12)	0.0096 (12)
O13D	0.0395 (16)	0.0211 (13)	0.0236 (15)	−0.0104 (11)	0.0004 (12)	−0.0002 (11)
O14A	0.0231 (14)	0.0243 (13)	0.0240 (15)	0.0021 (10)	−0.0040 (11)	0.0073 (11)
O14B	0.0322 (15)	0.0301 (14)	0.0172 (15)	−0.0058 (11)	−0.0049 (12)	0.0007 (11)
O14C	0.0398 (17)	0.0380 (15)	0.0211 (15)	−0.0043 (12)	−0.0105 (13)	0.0050 (12)
O14D	0.0345 (15)	0.0229 (13)	0.0223 (15)	−0.0099 (11)	0.0053 (11)	0.0050 (11)
O21A	0.111 (3)	0.0417 (18)	0.030 (2)	−0.0270 (17)	−0.0076 (18)	0.0036 (16)
O21B	0.080 (2)	0.0224 (15)	0.0413 (19)	0.0054 (15)	0.0028 (16)	−0.0052 (14)
O21C	0.102 (3)	0.0254 (16)	0.0350 (19)	0.0044 (16)	−0.0021 (17)	−0.0048 (14)
O21D	0.063 (2)	0.0326 (16)	0.0276 (18)	0.0078 (14)	−0.0054 (15)	−0.0039 (14)
O22A	0.171 (4)	0.0407 (19)	0.048 (2)	−0.039 (2)	0.001 (2)	0.0168 (18)
O22B	0.070 (2)	0.0582 (19)	0.0254 (18)	−0.0220 (16)	−0.0124 (16)	−0.0147 (15)
O22C	0.069 (2)	0.064 (2)	0.0262 (18)	−0.0232 (16)	−0.0142 (16)	−0.0139 (15)
O22D	0.080 (2)	0.0337 (17)	0.054 (2)	−0.0240 (16)	0.0004 (17)	0.0134 (16)
N2A	0.039 (2)	0.0297 (19)	0.029 (2)	−0.0024 (15)	−0.0024 (17)	0.0106 (17)
N2B	0.043 (2)	0.0255 (18)	0.025 (2)	−0.0122 (16)	0.0077 (16)	−0.0063 (15)
N2C	0.050 (2)	0.0299 (19)	0.021 (2)	−0.0138 (16)	−0.0002 (17)	−0.0022 (16)
N2D	0.036 (2)	0.0257 (18)	0.036 (2)	−0.0021 (15)	−0.0112 (17)	0.0012 (17)
C1A	0.0126 (18)	0.0249 (19)	0.018 (2)	−0.0005 (15)	0.0001 (15)	−0.0033 (16)
C1B	0.0114 (18)	0.0205 (18)	0.023 (2)	−0.0037 (14)	−0.0024 (15)	0.0007 (16)
C1C	0.0125 (18)	0.0206 (18)	0.019 (2)	−0.0029 (14)	−0.0006 (15)	−0.0003 (16)
C1D	0.0155 (19)	0.0248 (19)	0.019 (2)	−0.0026 (15)	0.0006 (15)	0.0004 (16)
C2A	0.0169 (19)	0.027 (2)	0.023 (2)	−0.0024 (15)	−0.0009 (16)	0.0035 (17)
C2B	0.0195 (19)	0.0168 (18)	0.021 (2)	−0.0050 (14)	0.0026 (16)	−0.0031 (16)
C2C	0.020 (2)	0.0184 (18)	0.022 (2)	−0.0045 (15)	−0.0009 (16)	−0.0026 (16)
C2D	0.0162 (19)	0.0226 (19)	0.026 (2)	−0.0039 (15)	0.0000 (16)	−0.0004 (17)
C3A	0.025 (2)	0.044 (2)	0.024 (2)	−0.0049 (18)	−0.0043 (17)	0.0118 (19)
C3B	0.027 (2)	0.029 (2)	0.023 (2)	−0.0048 (17)	−0.0016 (17)	0.0025 (18)
C3C	0.029 (2)	0.034 (2)	0.023 (2)	−0.0042 (17)	−0.0060 (17)	0.0082 (18)
C3D	0.027 (2)	0.037 (2)	0.023 (2)	−0.0041 (17)	−0.0032 (17)	0.0080 (19)
C4A	0.030 (2)	0.059 (3)	0.017 (2)	−0.007 (2)	−0.0052 (18)	0.000 (2)
C4B	0.030 (2)	0.020 (2)	0.037 (3)	−0.0017 (16)	−0.0011 (19)	0.0083 (18)
C4C	0.029 (2)	0.023 (2)	0.037 (3)	−0.0025 (16)	−0.0012 (19)	0.0110 (19)
C4D	0.032 (2)	0.047 (3)	0.024 (2)	−0.0042 (19)	−0.0071 (19)	−0.002 (2)
C5A	0.026 (2)	0.041 (2)	0.027 (2)	−0.0059 (18)	−0.0018 (18)	−0.0081 (19)
C5B	0.024 (2)	0.0181 (19)	0.033 (2)	−0.0024 (15)	0.0013 (17)	−0.0047 (17)
C5C	0.021 (2)	0.0195 (19)	0.045 (3)	−0.0028 (16)	0.0013 (18)	−0.0041 (18)
C5D	0.029 (2)	0.035 (2)	0.031 (3)	−0.0053 (17)	−0.0042 (19)	−0.0110 (19)
C6A	0.022 (2)	0.028 (2)	0.022 (2)	0.0002 (16)	−0.0016 (16)	−0.0006 (17)
C6B	0.019 (2)	0.026 (2)	0.022 (2)	−0.0042 (15)	−0.0010 (16)	−0.0046 (16)
C6C	0.0179 (19)	0.028 (2)	0.024 (2)	−0.0050 (15)	−0.0030 (16)	−0.0039 (17)
C6D	0.025 (2)	0.024 (2)	0.028 (2)	−0.0018 (16)	−0.0015 (17)	0.0020 (17)
C11A	0.0198 (19)	0.0247 (19)	0.015 (2)	−0.0050 (15)	−0.0008 (15)	0.0017 (16)
C11B	0.022 (2)	0.0200 (19)	0.021 (2)	−0.0055 (15)	0.0020 (16)	−0.0028 (16)
C11C	0.0167 (19)	0.0192 (18)	0.025 (2)	−0.0026 (14)	0.0002 (16)	−0.0025 (16)
C11D	0.021 (2)	0.0195 (19)	0.025 (2)	0.0005 (15)	−0.0016 (16)	−0.0037 (16)
C12A	0.022 (2)	0.0242 (19)	0.022 (2)	−0.0004 (15)	0.0022 (16)	−0.0004 (17)
C12B	0.032 (2)	0.0235 (19)	0.019 (2)	−0.0099 (16)	−0.0027 (17)	−0.0006 (16)
C12C	0.022 (2)	0.028 (2)	0.026 (2)	−0.0066 (16)	−0.0055 (17)	0.0029 (17)
C12D	0.026 (2)	0.030 (2)	0.023 (2)	−0.0122 (16)	−0.0017 (17)	−0.0004 (18)
C13A	0.0158 (19)	0.024 (2)	0.021 (2)	−0.0053 (15)	−0.0011 (15)	−0.0006 (17)
C13B	0.021 (2)	0.0200 (19)	0.016 (2)	0.0002 (15)	0.0043 (16)	−0.0019 (16)
C13C	0.024 (2)	0.030 (2)	0.019 (2)	0.0055 (17)	0.0007 (17)	−0.0018 (17)
C13D	0.0162 (19)	0.024 (2)	0.021 (2)	−0.0009 (15)	0.0035 (15)	0.0013 (17)

### Geometric parameters (Å, °)

**Table:**

Rb1—O1W	2.922 (2)	C1A—C6A	1.394 (5)
Rb1—O2W	2.849 (2)	C1A—C2A	1.396 (5)
Rb1—O3W	2.952 (2)	C1B—C6B	1.385 (4)
Rb1—O4W	3.106 (2)	C1B—C2B	1.389 (5)
Rb1—O13A	2.867 (2)	C1B—C11B	1.472 (4)
Rb1—O14D^i^	2.889 (2)	C1C—C11C	1.480 (4)
Rb1—O14B^ii^	2.870 (2)	C1C—C2C	1.395 (5)
Rb2—O4W	2.986 (2)	C1C—C6C	1.385 (4)
Rb2—O5W	2.927 (2)	C1D—C2D	1.397 (5)
Rb2—O6W	3.132 (2)	C1D—C6D	1.394 (5)
Rb2—O14C	3.031 (2)	C1D—C11D	1.470 (5)
Rb2—O3W^iii^	3.047 (2)	C2A—C3A	1.386 (5)
Rb2—O14A^iii^	2.908 (2)	C2B—C3B	1.383 (5)
Rb2—O13D^i^	2.977 (2)	C2C—C3C	1.382 (5)
Rb3—O2W	2.975 (2)	C2D—C3D	1.379 (5)
Rb3—O13B	2.952 (2)	C3A—C4A	1.365 (5)
Rb3—O13D	2.926 (2)	C3B—C4B	1.378 (5)
Rb3—O21D	2.944 (3)	C3C—C4C	1.377 (5)
Rb3—O14D^i^	2.901 (2)	C3D—C4D	1.370 (5)
Rb3—O14B^ii^	2.947 (2)	C4A—C5A	1.384 (5)
Rb4—O13A	3.018 (2)	C4B—C5B	1.386 (5)
Rb4—O21A	2.969 (3)	C4C—C5C	1.386 (6)
Rb4—O14A^iv^	2.955 (2)	C4D—C5D	1.385 (5)
Rb4—O14C^iv^	2.889 (2)	C5A—C6A	1.374 (5)
Rb4—O5W^v^	3.190 (2)	C5B—C6B	1.385 (5)
Rb4—O13C^v^	2.883 (2)	C5C—C6C	1.374 (5)
O13A—C13A	1.258 (4)	C5D—C6D	1.377 (5)
O13B—C13B	1.247 (4)	C11A—C12A	1.322 (5)
O13C—C13C	1.256 (4)	C11B—C12B	1.326 (5)
O13D—C13D	1.256 (4)	C11C—C12C	1.312 (5)
O14A—C13A	1.273 (4)	C11D—C12D	1.320 (5)
O14B—C13B	1.281 (4)	C12A—C13A	1.487 (5)
O14C—C13C	1.262 (4)	C12B—C13B	1.483 (4)
O14D—C13D	1.261 (4)	C12C—C13C	1.497 (5)
O21A—N2A	1.204 (5)	C12D—C13D	1.490 (5)
O21B—N2B	1.228 (4)	C3A—H3A	0.9500
O21C—N2C	1.231 (4)	C3B—H3B	0.9500
O21D—N2D	1.223 (5)	C3C—H3C	0.9500
O22A—N2A	1.210 (5)	C3D—H3D	0.9500
O22B—N2B	1.220 (4)	C4A—H4A	0.9500
O22C—N2C	1.220 (4)	C4B—H4B	0.9500
O22D—N2D	1.219 (4)	C4C—H4C	0.9500
O1W—H12W	0.8600	C4D—H4D	0.9500
O1W—H11W	0.8700	C5A—H5A	0.9500
O2W—H21W	0.8200	C5B—H5B	0.9500
O2W—H22W	0.8500	C5C—H5C	0.9500
O3W—H32W	0.8800	C5D—H5D	0.9500
O3W—H31W	0.9100	C6A—H6A	0.9500
O4W—H42W	0.8400	C6B—H6B	0.9500
O4W—H41W	0.8400	C6C—H6C	0.9500
O5W—H51W	0.9400	C6D—H6D	0.9500
O5W—H52W	0.8300	C11A—H11A	0.9500
O6W—H61W	0.8600	C11B—H11B	0.9500
O6W—H62W	0.8500	C11C—H11C	0.9500
N2A—C2A	1.476 (5)	C11D—H11D	0.9500
N2B—C2B	1.483 (4)	C12A—H12A	0.9500
N2C—C2C	1.473 (4)	C12B—H12B	0.9500
N2D—C2D	1.478 (5)	C12C—H12C	0.9500
C1A—C11A	1.476 (5)	C12D—H12D	0.9500
			
O1W—Rb1—O2W	162.58 (7)	O21C—N2C—O22C	123.6 (3)
O1W—Rb1—O3W	87.71 (6)	O21D—N2D—C2D	119.0 (3)
O1W—Rb1—O4W	56.37 (6)	O22D—N2D—C2D	117.5 (3)
O1W—Rb1—O13A	116.20 (7)	O21D—N2D—O22D	123.6 (3)
O1W—Rb1—O14D^i^	86.68 (6)	C2A—C1A—C6A	115.6 (3)
O1W—Rb1—O14B^ii^	107.58 (7)	C6A—C1A—C11A	118.4 (3)
O2W—Rb1—O3W	107.75 (6)	C2A—C1A—C11A	126.0 (3)
O2W—Rb1—O4W	123.12 (6)	C2B—C1B—C6B	115.6 (3)
O2W—Rb1—O13A	76.13 (7)	C6B—C1B—C11B	120.7 (3)
O2W—Rb1—O14D^i^	76.65 (7)	C2B—C1B—C11B	123.5 (3)
O2W—Rb1—O14B^ii^	66.82 (7)	C2C—C1C—C11C	124.2 (3)
O3W—Rb1—O4W	107.40 (6)	C6C—C1C—C11C	120.3 (3)
O3W—Rb1—O13A	77.01 (6)	C2C—C1C—C6C	115.5 (3)
O3W—Rb1—O14D^i^	168.43 (7)	C6D—C1D—C11D	119.4 (3)
O3W—Rb1—O14B^ii^	85.81 (6)	C2D—C1D—C6D	115.2 (3)
O4W—Rb1—O13A	70.06 (7)	C2D—C1D—C11D	125.4 (3)
O4W—Rb1—O14D^i^	77.69 (6)	N2A—C2A—C3A	116.2 (3)
O4W—Rb1—O14B^ii^	157.51 (7)	C1A—C2A—C3A	122.4 (3)
O13A—Rb1—O14D^i^	114.55 (6)	N2A—C2A—C1A	121.3 (3)
O13A—Rb1—O14B^ii^	131.78 (7)	N2B—C2B—C1B	120.7 (3)
O14B^ii^—Rb1—O14D^i^	86.25 (6)	C1B—C2B—C3B	123.8 (3)
O4W—Rb2—O5W	113.24 (6)	N2B—C2B—C3B	115.5 (3)
O4W—Rb2—O6W	87.01 (6)	N2C—C2C—C1C	120.5 (3)
O4W—Rb2—O14C	75.46 (6)	C1C—C2C—C3C	123.4 (3)
O3W^iii^—Rb2—O4W	97.87 (6)	N2C—C2C—C3C	116.1 (3)
O4W—Rb2—O14A^iii^	169.11 (7)	N2D—C2D—C3D	116.4 (3)
O4W—Rb2—O13D^i^	69.65 (6)	N2D—C2D—C1D	120.0 (3)
O5W—Rb2—O6W	159.69 (6)	C1D—C2D—C3D	123.6 (3)
O5W—Rb2—O14C	87.92 (6)	C2A—C3A—C4A	120.1 (3)
O3W^iii^—Rb2—O5W	122.39 (6)	C2B—C3B—C4B	118.5 (3)
O5W—Rb2—O14A^iii^	73.41 (6)	C2C—C3C—C4C	119.1 (3)
O5W—Rb2—O13D^i^	79.29 (7)	C2D—C3D—C4D	119.2 (3)
O6W—Rb2—O14C	96.02 (6)	C3A—C4A—C5A	119.3 (3)
O3W^iii^—Rb2—O6W	52.37 (6)	C3B—C4B—C5B	120.1 (3)
O6W—Rb2—O14A^iii^	86.33 (6)	C3C—C4C—C5C	119.1 (3)
O6W—Rb2—O13D^i^	111.05 (6)	C3D—C4D—C5D	119.4 (3)
O3W^iii^—Rb2—O14C	148.30 (6)	C4A—C5A—C6A	120.2 (3)
O14A^iii^—Rb2—O14C	96.69 (6)	C4B—C5B—C6B	119.5 (3)
O13D^i^—Rb2—O14C	133.82 (6)	C4C—C5C—C6C	120.4 (3)
O3W^iii^—Rb2—O14A^iii^	84.84 (6)	C4D—C5D—C6D	120.5 (3)
O3W^iii^—Rb2—O13D^i^	67.50 (6)	C1A—C6A—C5A	122.4 (3)
O13D^i^—Rb2—O14A^iii^	120.90 (6)	C1B—C6B—C5B	122.6 (3)
O2W—Rb3—O13B	137.47 (6)	C1C—C6C—C5C	122.5 (3)
O2W—Rb3—O13D	90.66 (7)	C1D—C6D—C5D	122.2 (3)
O2W—Rb3—O21D	146.59 (7)	C1A—C11A—C12A	125.0 (3)
O2W—Rb3—O14D^i^	74.53 (7)	C1B—C11B—C12B	125.4 (3)
O2W—Rb3—O14B^ii^	64.25 (6)	C1C—C11C—C12C	123.8 (3)
O13B—Rb3—O13D	78.95 (6)	C1D—C11D—C12D	124.1 (3)
O13B—Rb3—O21D	75.95 (7)	C11A—C12A—C13A	123.0 (3)
O13B—Rb3—O14D^i^	100.93 (6)	C11B—C12B—C13B	123.8 (3)
O13B—Rb3—O14B^ii^	158.27 (7)	C11C—C12C—C13C	124.4 (3)
O13D—Rb3—O21D	98.02 (7)	C11D—C12D—C13D	124.8 (3)
O13D—Rb3—O14D^i^	158.10 (7)	O14A—C13A—C12A	116.7 (3)
O13D—Rb3—O14B^ii^	103.66 (6)	O13A—C13A—O14A	124.0 (3)
O14D^i^—Rb3—O21D	103.21 (7)	O13A—C13A—C12A	119.2 (3)
O14B^ii^—Rb3—O21D	82.34 (7)	O14B—C13B—C12B	115.9 (3)
O14B^ii^—Rb3—O14D^i^	84.63 (6)	O13B—C13B—O14B	123.7 (3)
O13A—Rb4—O21A	91.50 (7)	O13B—C13B—C12B	120.3 (3)
O13A—Rb4—O14A^iv^	156.07 (7)	O14C—C13C—C12C	115.5 (3)
O13A—Rb4—O14C^iv^	82.00 (6)	O13C—C13C—C12C	119.6 (3)
O5W^v^—Rb4—O13A	96.67 (6)	O13C—C13C—O14C	124.9 (3)
O13A—Rb4—O13C^v^	106.82 (6)	O13D—C13D—C12D	119.0 (3)
O14A^iv^—Rb4—O21A	105.01 (7)	O14D—C13D—C12D	115.7 (3)
O14C^iv^—Rb4—O21A	73.64 (8)	O13D—C13D—O14D	125.3 (3)
O5W^v^—Rb4—O21A	170.09 (7)	C2A—C3A—H3A	120.00
O13C^v^—Rb4—O21A	79.63 (8)	C4A—C3A—H3A	120.00
O14A^iv^—Rb4—O14C^iv^	86.13 (6)	C2B—C3B—H3B	121.00
O5W^v^—Rb4—O14A^iv^	69.04 (6)	C4B—C3B—H3B	121.00
O13C^v^—Rb4—O14A^iv^	93.32 (6)	C2C—C3C—H3C	120.00
O5W^v^—Rb4—O14C^iv^	112.98 (6)	C4C—C3C—H3C	121.00
O13C^v^—Rb4—O14C^iv^	152.12 (7)	C2D—C3D—H3D	120.00
O5W^v^—Rb4—O13C^v^	92.60 (6)	C4D—C3D—H3D	120.00
Rb1—O2W—Rb3	88.47 (7)	C3A—C4A—H4A	120.00
Rb1—O3W—Rb2^ii^	96.96 (6)	C5A—C4A—H4A	120.00
Rb1—O4W—Rb2	105.80 (7)	C3B—C4B—H4B	120.00
Rb2—O5W—Rb4^vi^	91.75 (7)	C5B—C4B—H4B	120.00
Rb1—O13A—Rb4	93.79 (7)	C3C—C4C—H4C	121.00
Rb1—O13A—C13A	124.9 (2)	C5C—C4C—H4C	120.00
Rb4—O13A—C13A	123.3 (2)	C3D—C4D—H4D	120.00
Rb3—O13B—C13B	111.2 (2)	C5D—C4D—H4D	120.00
Rb4^vi^—O13C—C13C	115.8 (2)	C4A—C5A—H5A	120.00
Rb3—O13D—C13D	127.7 (2)	C6A—C5A—H5A	120.00
Rb2^iv^—O13D—Rb3	91.02 (7)	C4B—C5B—H5B	120.00
Rb2^iv^—O13D—C13D	124.6 (2)	C6B—C5B—H5B	120.00
Rb4^i^—O14A—C13A	134.0 (2)	C4C—C5C—H5C	120.00
Rb2^ii^—O14A—C13A	123.9 (2)	C6C—C5C—H5C	120.00
Rb2^ii^—O14A—Rb4^i^	97.10 (7)	C4D—C5D—H5D	120.00
Rb1^iii^—O14B—C13B	122.85 (19)	C6D—C5D—H5D	120.00
Rb3^iii^—O14B—C13B	116.9 (2)	C1A—C6A—H6A	119.00
Rb1^iii^—O14B—Rb3^iii^	88.60 (6)	C5A—C6A—H6A	119.00
Rb2—O14C—C13C	104.89 (19)	C1B—C6B—H6B	119.00
Rb2—O14C—Rb4^i^	121.16 (8)	C5B—C6B—H6B	119.00
Rb4^i^—O14C—C13C	129.2 (2)	C1C—C6C—H6C	119.00
Rb1^iv^—O14D—C13D	119.1 (2)	C5C—C6C—H6C	119.00
Rb3^iv^—O14D—C13D	139.7 (2)	C1D—C6D—H6D	119.00
Rb1^iv^—O14D—Rb3^iv^	89.15 (6)	C5D—C6D—H6D	119.00
Rb4—O21A—N2A	164.5 (3)	C1A—C11A—H11A	117.00
Rb3—O21D—N2D	149.2 (2)	C12A—C11A—H11A	118.00
H11W—O1W—H12W	100.00	C1B—C11B—H11B	117.00
H21W—O2W—H22W	109.00	C12B—C11B—H11B	117.00
H31W—O3W—H32W	108.00	C1C—C11C—H11C	118.00
H41W—O4W—H42W	103.00	C12C—C11C—H11C	118.00
H51W—O5W—H52W	103.00	C1D—C11D—H11D	118.00
H61W—O6W—H62W	103.00	C12D—C11D—H11D	118.00
O22A—N2A—C2A	117.8 (3)	C11A—C12A—H12A	119.00
O21A—N2A—O22A	121.4 (3)	C13A—C12A—H12A	118.00
O21A—N2A—C2A	120.8 (3)	C11B—C12B—H12B	118.00
O22B—N2B—C2B	118.3 (3)	C13B—C12B—H12B	118.00
O21B—N2B—C2B	117.7 (3)	C11C—C12C—H12C	118.00
O21B—N2B—O22B	124.0 (3)	C13C—C12C—H12C	118.00
O21C—N2C—C2C	118.0 (3)	C11D—C12D—H12D	118.00
O22C—N2C—C2C	118.3 (3)	C13D—C12D—H12D	118.00
			
O3W—Rb1—O2W—Rb3	−128.58 (6)	O14C^iv^—Rb4—O13A—C13A	130.7 (3)
O4W—Rb1—O2W—Rb3	105.70 (7)	O5W^v^—Rb4—O13A—C13A	−116.9 (3)
O13A—Rb1—O2W—Rb3	160.21 (7)	O14A^iv^—Rb4—O13A—Rb1	−31.47 (18)
O14D^i^—Rb1—O2W—Rb3	40.31 (6)	O13C^v^—Rb4—O13A—Rb1	114.66 (7)
O14B^ii^—Rb1—O2W—Rb3	−51.33 (6)	O14A^iii^—Rb4^vi^—O5W—Rb2	−38.40 (6)
O1W^iv^—Rb1^iv^—O14D—C13D	−16.4 (2)	O13A^vi^—Rb4^vi^—O5W—Rb2	161.44 (6)
O2W^iv^—Rb1^iv^—O14D—C13D	168.7 (2)	O5W^ii^—Rb4^i^—O14A—C13A	−166.8 (3)
O4W^iv^—Rb1^iv^—O14D—C13D	39.9 (2)	O13C^ii^—Rb4^i^—O14A—C13A	101.7 (3)
O13A^iv^—Rb1^iv^—O14D—C13D	101.0 (2)	O14C^iv^—Rb4—O13A—Rb1	−92.46 (7)
O14B^v^—Rb1^iv^—O14D—C13D	−124.3 (2)	O5W—Rb4^vi^—O13C—C13C	−52.3 (2)
O1W—Rb1—O3W—Rb2^ii^	47.34 (7)	O13A^vi^—Rb4^vi^—O13C—C13C	−150.1 (2)
O2W—Rb1—O3W—Rb2^ii^	−140.82 (7)	O13A^i^—Rb4^i^—O14A—C13A	−110.6 (3)
O4W—Rb1—O3W—Rb2^ii^	−6.26 (8)	O14A^iii^—Rb4^vi^—O13C—C13C	16.8 (2)
O13A—Rb1—O3W—Rb2^ii^	−70.21 (7)	O21A^i^—Rb4^i^—O14A—C13A	21.6 (3)
O14B^ii^—Rb1—O3W—Rb2^ii^	155.16 (7)	O14C—Rb4^i^—O14A—Rb2^ii^	155.45 (8)
O14B^ii^—Rb1—O13A—Rb4	9.85 (10)	O13C^ii^—Rb4^i^—O14C—Rb2	−129.00 (13)
O1W—Rb1—O13A—C13A	−7.4 (3)	O21A—Rb4—O13A—Rb1	−165.71 (8)
O2W—Rb1—O13A—C13A	−174.2 (3)	O14A—Rb4^i^—O14C—Rb2	−39.22 (9)
O3W—Rb1—O13A—C13A	73.5 (2)	O13C—Rb4^vi^—O5W—Rb2	54.16 (7)
O4W—Rb1—O13A—C13A	−40.7 (2)	O14C—Rb4^i^—O14A—C13A	−50.4 (3)
O1W—Rb1—O4W—Rb2	140.01 (10)	Rb4—O13A—C13A—O14A	128.4 (3)
O2W—Rb1—O4W—Rb2	−19.11 (11)	Rb1—O13A—C13A—O14A	4.7 (5)
O3W—Rb1—O4W—Rb2	−144.98 (7)	Rb1—O13A—C13A—C12A	−175.1 (2)
O13A—Rb1—O4W—Rb2	−76.35 (8)	Rb4—O13A—C13A—C12A	−51.5 (4)
O14D^i^—Rb1—O4W—Rb2	45.77 (7)	Rb3—O13B—C13B—O14B	−81.9 (3)
O14B^ii^—Rb1—O4W—Rb2	91.20 (17)	Rb3—O13B—C13B—C12B	96.5 (3)
O1W—Rb1—O13A—Rb4	−143.14 (6)	Rb4^vi^—O13C—C13C—O14C	88.2 (4)
O2W—Rb1—O13A—Rb4	50.00 (6)	Rb4^vi^—O13C—C13C—C12C	−90.9 (3)
O14D^vi^—Rb1^iii^—O14B—C13B	96.4 (2)	Rb2^iv^—O13D—C13D—C12D	−161.1 (2)
O1W^iii^—Rb1^iii^—O14B—C13B	11.1 (2)	Rb3—O13D—C13D—O14D	143.9 (3)
O2W^iii^—Rb1^iii^—O14B—C13B	173.5 (3)	Rb3—O13D—C13D—C12D	−37.1 (4)
O3W^iii^—Rb1^iii^—O14B—C13B	−75.2 (2)	Rb2^iv^—O13D—C13D—O14D	19.8 (5)
O4W^iii^—Rb1^iii^—O14B—C13B	52.2 (3)	Rb2^ii^—O14A—C13A—C12A	106.7 (3)
O13A^iii^—Rb1^iii^—O14B—C13B	−143.6 (2)	Rb2^ii^—O14A—C13A—O13A	−73.1 (4)
O14B^ii^—Rb1—O13A—C13A	145.6 (2)	Rb4^i^—O14A—C13A—O13A	138.2 (3)
O14D^i^—Rb1—O13A—Rb4	118.02 (6)	Rb4^i^—O14A—C13A—C12A	−41.9 (4)
O14D^i^—Rb1—O13A—C13A	−106.2 (2)	Rb3^iii^—O14B—C13B—C12B	67.8 (3)
O3W—Rb1—O13A—Rb4	−62.29 (6)	Rb3^iii^—O14B—C13B—O13B	−113.8 (3)
O4W—Rb1—O13A—Rb4	−176.51 (7)	Rb1^iii^—O14B—C13B—O13B	139.1 (3)
O6W—Rb2—O14C—C13C	−76.6 (2)	Rb1^iii^—O14B—C13B—C12B	−39.3 (4)
O3W^iii^—Rb2—O5W—Rb4^vi^	110.48 (7)	Rb4^i^—O14C—C13C—O13C	101.8 (4)
O14A^iii^—Rb2—O5W—Rb4^vi^	37.95 (6)	Rb2—O14C—C13C—O13C	−103.0 (3)
O6W—Rb2—O4W—Rb1	42.82 (7)	Rb2—O14C—C13C—C12C	76.2 (3)
O3W^iii^—Rb2—O14C—C13C	−80.6 (2)	Rb4^i^—O14C—C13C—C12C	−79.1 (3)
O5W—Rb2—O4W—Rb1	−138.91 (7)	Rb1^iv^—O14D—C13D—O13D	−91.9 (4)
O5W—Rb2—O14C—C13C	83.5 (2)	Rb1^iv^—O14D—C13D—C12D	89.0 (3)
O14A—Rb2^ii^—O3W—Rb1	44.45 (7)	Rb3^iv^—O14D—C13D—O13D	139.2 (3)
O4W^ii^—Rb2^ii^—O3W—Rb1	−146.17 (7)	Rb3^iv^—O14D—C13D—C12D	−39.9 (5)
O5W^ii^—Rb2^ii^—O3W—Rb1	−22.17 (10)	Rb3—O21D—N2D—O22D	85.3 (5)
O6W^ii^—Rb2^ii^—O3W—Rb1	133.79 (9)	Rb3—O21D—N2D—C2D	−95.8 (5)
O14C^ii^—Rb2^ii^—O3W—Rb1	138.82 (9)	O21A—N2A—C2A—C1A	−15.1 (5)
O14C^iv^—Rb2^iv^—O13D—Rb3	−32.16 (10)	O21A—N2A—C2A—C3A	169.4 (3)
O4W—Rb2—O14C—C13C	−161.9 (2)	O22A—N2A—C2A—C1A	161.9 (4)
O14C^ii^—Rb2^ii^—O14A—C13A	−114.0 (3)	O22A—N2A—C2A—C3A	−13.5 (5)
O3W—Rb2^ii^—O14A—Rb4^i^	−167.99 (7)	O21B—N2B—C2B—C1B	36.3 (5)
O6W^iv^—Rb2^iv^—O13D—C13D	−14.8 (3)	O21B—N2B—C2B—C3B	−141.2 (3)
O14C^iv^—Rb2^iv^—O13D—C13D	106.9 (2)	O22B—N2B—C2B—C1B	−146.7 (3)
O14C—Rb2—O5W—Rb4^vi^	−59.66 (6)	O22B—N2B—C2B—C3B	35.8 (5)
O5W^iv^—Rb2^iv^—O13D—Rb3	44.39 (6)	O21C—N2C—C2C—C1C	−32.8 (5)
O14C—Rb2—O4W—Rb1	139.84 (8)	O21C—N2C—C2C—C3C	144.2 (4)
O3W^iii^—Rb2—O4W—Rb1	−8.54 (8)	O22C—N2C—C2C—C1C	150.4 (3)
O13D^i^—Rb2—O4W—Rb1	−70.94 (7)	O22C—N2C—C2C—C3C	−32.5 (5)
O4W—Rb2—O5W—Rb4^vi^	−132.86 (6)	O21D—N2D—C2D—C1D	34.7 (5)
O6W—Rb2—O5W—Rb4^vi^	42.1 (2)	O21D—N2D—C2D—C3D	−145.6 (4)
O13D^i^—Rb2—O5W—Rb4^vi^	164.94 (7)	O22D—N2D—C2D—C1D	−146.3 (3)
O3W^iii^—Rb2—O14C—Rb4^i^	77.21 (15)	O22D—N2D—C2D—C3D	33.4 (5)
O6W^iv^—Rb2^iv^—O13D—Rb3	−153.83 (5)	C6A—C1A—C2A—N2A	−172.4 (3)
O3W—Rb2^ii^—O14A—C13A	34.2 (3)	C6A—C1A—C2A—C3A	2.8 (5)
O4W^iv^—Rb2^iv^—O13D—C13D	63.5 (2)	C11A—C1A—C2A—N2A	10.3 (5)
O5W^iv^—Rb2^iv^—O13D—C13D	−176.6 (2)	C11A—C1A—C2A—C3A	−174.6 (3)
O5W—Rb2—O14C—Rb4^i^	−118.77 (10)	C2A—C1A—C6A—C5A	−2.3 (5)
O6W—Rb2—O14C—Rb4^i^	81.21 (9)	C11A—C1A—C6A—C5A	175.3 (3)
O13D^i^—Rb2—O14C—C13C	156.45 (19)	C2A—C1A—C11A—C12A	−146.6 (4)
O14A^iii^—Rb2—O14C—Rb4^i^	168.21 (9)	C6A—C1A—C11A—C12A	36.1 (5)
O14A^iii^—Rb2—O14C—C13C	10.5 (2)	C6B—C1B—C2B—N2B	−175.6 (3)
O13D^i^—Rb2—O14C—Rb4^i^	−45.79 (13)	C6B—C1B—C2B—C3B	1.7 (5)
O4W—Rb2—O14C—Rb4^i^	−4.11 (9)	C11B—C1B—C2B—N2B	9.5 (5)
O6W^ii^—Rb2^ii^—O14A—C13A	−18.3 (3)	C11B—C1B—C2B—C3B	−173.3 (3)
O4W^iv^—Rb2^iv^—O13D—Rb3	−75.51 (6)	C2B—C1B—C6B—C5B	0.5 (4)
O5W^ii^—Rb2^ii^—O14A—C13A	160.2 (3)	C11B—C1B—C6B—C5B	175.5 (3)
O14D^i^—Rb3—O13D—Rb2^iv^	−20.70 (19)	C2B—C1B—C11B—C12B	−157.3 (3)
O21D—Rb3—O13D—Rb2^iv^	173.56 (7)	C6B—C1B—C11B—C12B	28.0 (5)
O14D^i^—Rb3—O2W—Rb1	−40.57 (6)	C6C—C1C—C2C—N2C	177.2 (3)
O14B^ii^—Rb3—O2W—Rb1	50.90 (6)	C6C—C1C—C2C—C3C	0.4 (5)
O2W—Rb3—O13D—Rb2^iv^	25.92 (6)	C11C—C1C—C2C—N2C	−5.8 (5)
O13B—Rb3—O2W—Rb1	−129.95 (8)	C11C—C1C—C2C—C3C	177.3 (3)
O13B—Rb3—O13D—Rb2^iv^	−112.55 (7)	C2C—C1C—C6C—C5C	−1.8 (4)
O14B^ii^—Rb3—O13B—C13B	−128.4 (2)	C11C—C1C—C6C—C5C	−178.9 (3)
O14B^ii^—Rb3—O13D—C13D	−47.4 (3)	C2C—C1C—C11C—C12C	147.8 (4)
O14D^vi^—Rb3^iii^—O14B—C13B	−101.5 (2)	C6C—C1C—C11C—C12C	−35.4 (5)
O14B^ii^—Rb3—O13D—Rb2^iv^	89.54 (7)	C6D—C1D—C2D—N2D	177.8 (3)
O13B^iv^—Rb3^iv^—O14D—C13D	−46.4 (3)	C6D—C1D—C2D—C3D	−1.9 (5)
O13D^iv^—Rb3^iv^—O14D—C13D	−134.0 (3)	C11D—C1D—C2D—N2D	2.0 (5)
O21D^iv^—Rb3^iv^—O14D—C13D	31.5 (3)	C11D—C1D—C2D—C3D	−177.7 (3)
O14B^v^—Rb3^iv^—O14D—C13D	112.3 (3)	C2D—C1D—C6D—C5D	2.6 (5)
O21D—Rb3—O2W—Rb1	50.06 (13)	C11D—C1D—C6D—C5D	178.7 (3)
O13D—Rb3—O2W—Rb1	155.77 (6)	C2D—C1D—C11D—C12D	−150.3 (4)
O2W^iii^—Rb3^iii^—O14B—C13B	−176.9 (2)	C6D—C1D—C11D—C12D	34.1 (5)
O2W—Rb3—O13B—C13B	53.7 (2)	N2A—C2A—C3A—C4A	174.2 (3)
O13D—Rb3—O13B—C13B	132.4 (2)	C1A—C2A—C3A—C4A	−1.2 (5)
O21D—Rb3—O13B—C13B	−126.3 (2)	N2B—C2B—C3B—C4B	175.4 (3)
O13D^iii^—Rb3^iii^—O14B—C13B	99.0 (2)	C1B—C2B—C3B—C4B	−2.1 (5)
O14D^i^—Rb3—O13B—C13B	−25.3 (2)	N2C—C2C—C3C—C4C	−175.8 (3)
O2W—Rb3—O21D—N2D	170.1 (4)	C1C—C2C—C3C—C4C	1.2 (5)
O13B—Rb3—O21D—N2D	−9.9 (4)	N2D—C2D—C3D—C4D	−179.4 (3)
O2W^iv^—Rb3^iv^—O14D—C13D	177.1 (3)	C1D—C2D—C3D—C4D	0.3 (5)
O13B—Rb3—O13D—C13D	110.5 (3)	C2A—C3A—C4A—C5A	−1.1 (5)
O21D—Rb3—O13D—C13D	36.6 (3)	C2B—C3B—C4B—C5B	0.3 (5)
O14D^i^—Rb3—O13D—C13D	−157.7 (2)	C2C—C3C—C4C—C5C	−1.4 (5)
O14B^ii^—Rb3—O21D—N2D	169.3 (4)	C2D—C3D—C4D—C5D	0.7 (5)
O14D^i^—Rb3—O21D—N2D	−108.1 (4)	C3A—C4A—C5A—C6A	1.7 (5)
O21D^iii^—Rb3^iii^—O14B—C13B	2.6 (2)	C3B—C4B—C5B—C6B	1.7 (5)
O13D—Rb3—O21D—N2D	66.5 (4)	C3C—C4C—C5C—C6C	0.1 (5)
O13B^iii^—Rb3^iii^—O14B—C13B	4.6 (3)	C3D—C4D—C5D—C6D	0.0 (5)
O2W—Rb3—O13D—C13D	−111.0 (3)	C4A—C5A—C6A—C1A	0.1 (5)
O13A^i^—Rb4^i^—O14C—C13C	−88.2 (3)	C4B—C5B—C6B—C1B	−2.1 (5)
O14A—Rb4^i^—O14C—C13C	112.6 (3)	C4C—C5C—C6C—C1C	1.6 (5)
O21A—Rb4—O13A—C13A	57.5 (3)	C4D—C5D—C6D—C1D	−1.8 (5)
O14C^iii^—Rb4^vi^—O5W—Rb2	−114.45 (7)	C1A—C11A—C12A—C13A	−175.4 (3)
O13A^i^—Rb4^i^—O14C—Rb2	119.95 (10)	C1B—C11B—C12B—C13B	−177.7 (3)
O21A^i^—Rb4^i^—O14C—Rb2	−146.10 (10)	C1C—C11C—C12C—C13C	175.5 (3)
O5W^ii^—Rb4^i^—O14C—Rb2	26.06 (11)	C1D—C11D—C12D—C13D	−174.4 (3)
O21A^i^—Rb4^i^—O14C—C13C	5.7 (2)	C11A—C12A—C13A—O13A	−8.7 (5)
O5W^ii^—Rb4^i^—O14C—C13C	177.9 (2)	C11A—C12A—C13A—O14A	171.5 (3)
O5W^v^—Rb4—O13A—Rb1	19.90 (6)	C11B—C12B—C13B—O13B	7.9 (5)
O13C^v^—Rb4—O13A—C13A	−22.2 (3)	C11B—C12B—C13B—O14B	−173.6 (3)
O21A^vi^—Rb4^vi^—O13C—C13C	121.5 (2)	C11C—C12C—C13C—O13C	−1.0 (5)
O13C^ii^—Rb4^i^—O14C—C13C	22.8 (3)	C11C—C12C—C13C—O14C	179.8 (3)
O14C^iii^—Rb4^vi^—O13C—C13C	104.8 (3)	C11D—C12D—C13D—O13D	−16.1 (5)
O14A^iv^—Rb4—O13A—C13A	−168.3 (2)	C11D—C12D—C13D—O14D	163.1 (3)

Symmetry codes: (i) *x*, *y*−1, *z*; (ii) *x*+1, *y*, *z*; (iii) *x*−1, *y*, *z*; (iv) *x*, *y*+1, *z*; (v) *x*+1, *y*+1, *z*; (vi) *x*−1, *y*−1, *z*.

### Hydrogen-bond geometry (Å, °)

**Table:**

*D*—H···*A*	*D*—H	H···*A*	*D*···*A*	*D*—H···*A*
O1W—H11W···O13B^vii^	0.87	1.94	2.795 (3)	167
O1W—H12W···O13D^vii^	0.86	1.91	2.753 (3)	167
O2W—H21W···O4W^iv^	0.82	1.97	2.788 (3)	170
O2W—H22W···O14C^iv^	0.85	1.93	2.716 (3)	153
O3W—H31W···O14D^vii^	0.91	1.80	2.695 (3)	169
O3W—H32W···O6W^ii^	0.88	1.86	2.728 (3)	170
O4W—H41W···O1W	0.84	2.02	2.852 (3)	178
O4W—H42W···O14A	0.84	1.91	2.758 (3)	180
O5W—H51W···O3W^vi^	0.94	1.82	2.734 (3)	163
O5W—H52W···O14B^i^	0.83	2.07	2.893 (3)	170
O6W—H61W···O13C^iv^	0.86	1.88	2.742 (3)	179
O6W—H62W···O13A	0.85	2.00	2.834 (3)	165
C4B—H4B···O21B^i^	0.95	2.56	3.290 (4)	134
C4C—H4C···O21C^iv^	0.95	2.58	3.290 (4)	132
C5A—H5A···O22C^viii^	0.95	2.57	3.238 (5)	128
C5D—H5D···O22B^ix^	0.95	2.55	3.252 (5)	131
C6D—H6D···O22D^iv^	0.95	2.60	3.297 (4)	131
C11A—H11A···O13A	0.95	2.51	2.826 (4)	100
C11A—H11A···O21A	0.95	2.29	2.741 (4)	108
C11B—H11B···O21B	0.95	2.47	2.819 (4)	102
C11D—H11D···O21D	0.95	2.46	2.801 (4)	101

Symmetry codes: (vii) *x*+1, *y*−1, *z*; (iv) *x*, *y*+1, *z*; (ii) *x*+1, *y*, *z*; (vi) *x*−1, *y*−1, *z*; (i) *x*, *y*−1, *z*; (viii) −*x*+1, −*y*+1, −*z*; (ix) −*x*+1, −*y*+3, −*z*+1.
